# Editor's Letter-Box

**Published:** 1899-08-19

**Authors:** 


### EDITOR'S LETTER-BOX.

[Our Correspondents are reminded that prolixity is a great bar to publication, and that brevity of style and conciseness of statement greatly facilitate early insertion.]

Dr. R. A. Birdwood, Medical Superintendent of the Park Hospital, Hither Green, writes: Kindly allow me to point out an error in the table you have directed attention to in the article on the North-Western Fever Hospital, and also to make these observations. The average daily number of patients at the North-Western is 371; that of staff 249. So that the ratio total staff to patients is 1 to 1·4, not 1 to 1 as stated in the reports. It is pointed out that the case mortality was high last year for each of the diseases at the North-Western. It is suggested that the small staff and high rate are related as cause to effect. During the preceding five years, however, 1893 to 1897, the North-Western had the lowest diphtheria death rate in 1893, and the lowest enteric fever rate in 1894 and 1896. On two occasions the diphtheria rate, and on four the scarlet and enteric fever rates, were below the average. It has repeatedly been claimed that the case mortality varies with the number of babies admitted. Table I. gives the diphtheria rates for all ages; Table V. the admissions at each hospital for various ages. At the North-Western 179 out of 890 admissions were under three years of age, say, 20 in 100; whereas at the South-Western, 60 out of 577, or 10 in 100, were below the same age. Only one hospital, the South-Eastern, exceeded the North-Western baby admission ratio, and that slightly, 21 in 100; whilst the South-Western had the fewest of all hospitals, the next to it being 13 in 100. If all admissions under five years of age are taken, then the North-Western has 43 in 100; whilst the South-Western has 32. Now the "all hospitals" diphtheria death-rate during 1898 for under three is 29·2, for under five is 23·7, and for all ages is 15·1. The fact is, the Board's reports supply ample material for independent inquiry.

As regards your remark about antitoxin, the point surely is not the number of patients in 100 to whom antitoxin is given, but the death-rate amongst those so treated. At the North-Western 433 had antitoxin—of that number 146 died, 33·7 in 100; whereas only 30 died out of 448 not injected. In 1894, before the introduction of antitoxin, 70 in 100 diphtheria patients admitted recovered. As to death-rate, there is no good reason for giving antitoxin to these 70.

The number of nurses employed in different hospitals does not vary altogether with the number of patients, but depends to a certain extent on the number of beds in a ward. A hospital with many small wards for the isolation and observation of patients requires more nurses than one in which there are few such wards. Any reduction in the death-rate brought about by nursing is at least as much due to the quality of the nursing as to the quantity. In this respect much good would result if the authorities of the great training schools for nurses would appreciate the value of experience gained in a fever hospital. I cannot understand how any nurse without such experience can be regarded as "fully trained." It is hopeless error to believe that a trained nurse must be a fool if she cannot nurse a scarlet fever or diphtheria patient without experience. Many well trained nurses have acknowledged to me that there is much to be learnt in a fever hospital after general training. If six to twelve months in a fever hospital formed part of a nurse's training a mutual advantage would result. The quality of nursing generally would be improved, and good as the nursing in the Board's hospitals is at present, there is room and welcome for others.

*** If, as Dr. Birdwood states, the figures given in the report of the Metropolitan Asylums Board are wrong, of course our criticism of these figures must be held in abeyance. It must be pointed out, however, that Dr. Birdwood's statement is itself a serious criticism of the report, for the figures referred to not only occur in a table, where perhaps an error might be overlooked, but are also specially quoted in a paragraph in the report summarising the table in question. We endeavoured not to burden our readers too greatly with statistics, but as Dr. Birdwood seems to suggests that the high mortality of the North-Western Hospital can be explained by the large proportion of its patients who are at an age when scarlet fever and diphtheria are peculiarly fatal, and especially draws attention to the number of cases admitted under three years of age, it may be well to refer to the actual figures. Taking scarlet fever first, we find that the cases under three years of age are 11·2 per cent. of the whole at the North-Western, against 10·5 at "all the hospitals," and 8·2 at the South-Western. But look at the difference in the mortality of these baby cases. North-Western, 15·2 per cent.; "all the hospitals," 14·8 per cent.; and South-Western, only 7·3. Dr. Birdwood objects to our comparing the South-Western with the North-Western because of its small number of babies, but here we find that, although the babies are comparatively few in number, their mortality is also small—less than half that at the North-Western. Turning now to diphtheria, we find that the proportions of infants under three years of age admitted at the different hospitals were—North-Western, 20·0 per cent.; all "the hospitals," 16·4; and South-Western only 10·3, as Dr. Birdwood points out. Truly a considerable difference. But, again, look at the difference in the mortality of these cases—North-Western, 34·0 per cent.; "all the hospitals," 29·2 per cent.; and South-Western, 20·0 per cent. Dr. Birdwood is quite right. The North-Western does have more than its share of babies, but it is not alone the abnormal number of these diphtheritic babies, but their abnormal mortality that swells the death-rate of this hospital. It is true that the North-Western has 3·6 per cent. more than its share of babies under three years old, but these North-Western babies die at a rate higher by 4·8 per cent. than that prevailing at "all the hospitals," and higher by 14 per cent. than that at the South-Western Hospital. We doubt whether dissecting the figures makes things much better for the North-Western. Dr. Birdwood refers to the fact that "only one hospital, the South-Eastern, exceeded the North-Western baby admission ratio," but he does not say, as he might have done, that the diphtheria mortality among these babies was 26·0 per cent., against 34·0 per cent. at the North-Western, nor does he point out that, notwithstanding this large proportion of baby admissions, the South-Eastern Hospital manages to show a far lower general death-rate in all diseases than occurs at the North-Western, so that excess of baby admissions cannot be the sole cause of the high death-rate of the latter hospital. In regard to the use of antitoxin we only introduced that as showing how distinctly the North-Western stands apart from the other hospitals of the Board, not merely in regard to results but also in regard to the only point in treatment of which any details are given. We think, as we said, that the figures given in the report are such as call for either inquiry or explanation. If the figures given in the report are wrong we fancy that some people will look on that as a still further reason for such a course.—Ed. *T. H.*

